# Rasd2 Mediates Acute Fasting-Induced Antidepressant-Like Effects via Dopamine D2 Receptor Activation in Ovariectomized Mice

**DOI:** 10.1093/ijnp/pyac082

**Published:** 2023-03-21

**Authors:** Ziqian Cheng, Chaohe Zhang, Fangyi Zhao, Jingjing Piao, Ranji Cui, Bingjin Li

**Affiliations:** Jilin Provincial Key Laboratory on Molecular and Chemical Genetic, Second Hospital of Jilin University, Changchun, P.R. China; Engineering Lab on screening of antidepressant drugs, Jilin Province Development and Reform Commission, Changchun, P.R. China; Jilin Provincial Key Laboratory on Molecular and Chemical Genetic, Second Hospital of Jilin University, Changchun, P.R. China; Engineering Lab on screening of antidepressant drugs, Jilin Province Development and Reform Commission, Changchun, P.R. China; Jilin Provincial Key Laboratory on Molecular and Chemical Genetic, Second Hospital of Jilin University, Changchun, P.R. China; Engineering Lab on screening of antidepressant drugs, Jilin Province Development and Reform Commission, Changchun, P.R. China; Jilin Provincial Key Laboratory on Molecular and Chemical Genetic, Second Hospital of Jilin University, Changchun, P.R. China; Engineering Lab on screening of antidepressant drugs, Jilin Province Development and Reform Commission, Changchun, P.R. China; Jilin Provincial Key Laboratory on Molecular and Chemical Genetic, Second Hospital of Jilin University, Changchun, P.R. China; Engineering Lab on screening of antidepressant drugs, Jilin Province Development and Reform Commission, Changchun, P.R. China; Jilin Provincial Key Laboratory on Molecular and Chemical Genetic, Second Hospital of Jilin University, Changchun, P.R. China; Engineering Lab on screening of antidepressant drugs, Jilin Province Development and Reform Commission, Changchun, P.R. China

**Keywords:** *Rasd2*, antidepressant, fasting, DRD2, RNA-seq

## Abstract

**Background:**

Previous studies have shown that estrogen and acute fasting for 9 hours have antidepressant-like effects by reducing immobility time in the forced swimming test. Estrogen and acute fasting share a common regulatory gene, *Rasd2*. RASD2 regulates dopamine D2 receptor (DRD2) transmission, but the role of *Rasd2* in the DRD2-mediated antidepressant-like effect of acute fasting has not been examined.

**Methods:**

In this study, open field test, forced swimming test, tail suspension test and sucrose preference test were used for behavioral assessments. RNA-seq, western blot, enzyme-linked immunosorbent assay, and co-immunoprecipitation were used to explore the role of *Rasd2* in a depression model induced by ovariectomy and the antidepressant-like effects of 9-hour fasting.

**Results:**

The RNA seq results showed that acute fasting induced a significant change in *Rasd2* gene expression. Depression-like behaviors induced by ovariectomy were associated with decreased RASD2 and DRD2 protein levels in the hippocampus, and *Rasd2* overexpression in the hippocampus alleviated depression-like behaviors and increased DRD2 expression. Nine-hour fasting had antidepressant-like effects in ovariectomized mice by upregulating the protein levels of RASD2, DRD2, CREB-BDNF, Akt, and estrogen receptor beta, and these effects can be blocked by DRD2 antagonists.

**Conclusions:**

Our results suggest that *Rasd2* and DRD2 play pivotal roles in depression-like behavior induced by ovariectomy. *Rasd2* regulates DRD2-mediated antidepressant-like effects of acute fasting in ovariectomized mice. *Rasd2* can therefore be postulated to be a potential therapeutic target for depression and perhaps also a potential predictive marker for depression.

**Significance Statement**

Previous studies have shown that estrogen and acute fasting for 9 hours have antidepressant-like effects by reducing immobility time in the forced swimming test. Estrogen and acute fasting share a common regulatory gene, *Rasd2*. RASD2 regulates dopamine D2 receptor (DRD2) transmission, but the role of *Rasd2* in the DRD2-mediated antidepressant-like effect of acute fasting has not been examined. In this study, behavioral assessments of antidepressant action were detected by open field test, forced swimming test, tail suspension test, and sucrose preference test. RNA-seq, western blot, enzyme-linked immunosorbent assay, and co-immunoprecipitation were used to explore the role of *Rasd2* in a depression model induced by ovariectomy and the antidepressant-like effects of 9-hour fasting. The present study suggests that *Rasd2* and DRD2 play pivotal roles in depression-like behavior induced by ovariectomy. *Rasd2* regulates DRD2-mediated antidepressant-like effects of acute fasting in ovariectomized mice. *Rasd2* can therefore be postulated to be a potential therapeutic target for depression and perhaps also a potential predictive marker for depression.

## INTRODUCTION

Depression is a mental disease characterized by low mood, psychomotor retardation, and cognitive impairment, which severely reduce quality of life (Nestler et al., 2002). Currently, depression is one of the leading causes of disability and a major contributor to the overall global burden of disease (Lancet, 2022). It is worth noting that a meta-analysis has shown that the heritability for major depression is approximately 37% (Flint and Kendler, 2014), and the prevalence of depression in women is almost twice that of men worldwide (Martin et al., 2013). In particular, women are at high risk of depression during hormonal transition phases (peripartum, perimenopause, etc.) (Freeman et al., 2014; Borgsted et al., 2022). However, preclinical study of depression in females remains understudied (Jiang et al., 2022; Lima et al., 2022).

Calorie restriction has been shown to extend the life span of several species over the past few decades (Fontana and Partridge, 2015) and has positive effects on neurological diseases, including Alzheimer’s disease and Parkinson’s disease (Zhang et al., 2021b; Ezzati and Pak, 2023; Govic et al., 2022). In our previous studies, mice treated with 9-hour fasting significantly shortened the immobility time of the forced swimming test (FST), whereas mice fasted for 3 hours and 18 hours had no significant changes (Li et al., 2014). Further studies revealed that acute fasting produces antidepressant-like effects through the activation of the cyclic adenosine monophosphate (cAMP)-response element binding protein (CREB)-brain-derived neurotrophic factor (BDNF) signaling pathway in the prefrontal cortex (PFC) and hippocampus (HP) (Lutter et al., 2008; Li et al., 2014; Cui et al., 2018; Wang et al., 2019). Additionally, caloric restriction upregulates estrogen receptor expression but has no effects on androgen receptor (Słuczanowska-Głąbowska et al., 2015). Fasting produces estrogenic effects in ovariectomized mice (Bigsby et al., 1997), and estrogen enhances the antidepressant-like effects of acute fasting via the activation of the CREB-BDNF signaling pathway in the PFC and HP (Wang et al., 2019). Therefore, fasting might be used as an adjunct to estrogen replacement therapy for depression.

RNA-seq data suggest that estrogen and acute fasting exert antidepressant-like effects through a common gene, *Rasd2* (Wang et al., 2019). Whether *Rasd2* participates in the antidepressant-like effects of fasting has not been directly examined yet, to our knowledge. RASD2 is a GTP binding protein that is highly enriched in the striatum and found at lower levels of expression in the HP, cerebral cortex, olfactory bulb, etc. (Vargiu et al., 2004). *Rasd2* negatively regulates G protein–coupled receptor-mediated cAMP production, and the targeted deletion of *Rasd2* in mice can significantly activate the cAMP/protein kinase A signaling pathway in the striatum (Vargiu et al., 2004; Errico et al., 2008; Ghiglieri et al., 2015). In addition, recent research indicates that *Rasd2* regulates the phosphoinositide 3-kinase (PI3K)/protein kinase B (Akt)/mechanistic target of rapamycin signaling pathway and consequently has a role in several neurological and psychiatric diseases, such as schizophrenia and Huntington’s disease (Emamian et al., 2004; Subramaniam et al., 2011; Lee et al., 2015). However, the role of *Rasd2* in depression remains unclear.

*Rasd2* function is closely tied to dopamine function. Depleting the striatum of dopaminergic input decreases *Rasd2* mRNA expression in the striatum (Harrison and LaHoste, 2006). In addition, activation of dopamine D2 receptors (DRD2) produces exaggerated stereotypy in *Rasd2* knockout mice (Quintero et al., 2008). Sciamanna et al. (2015) found that *Rasd2* deficiency produces aberrant DRD2-dependent activity through an abnormal Ca^2+^-dependent modulation of PI3K/Akt signaling. *Rasd2* mRNA has been located in dopamine D1 receptor-medium spiny neurons, DRD2-medium spiny neurons, and cholinergic interneurons (Sciamanna et al., 2015). *Rasd2* regulates dopamine-dependent neurotransmission by affecting the survival of nigrostriatal dopaminergic neurons (Sciamanna et al., 2015; Pinna et al., 2016). These findings suggest that *Rasd2* effects on other aspects of dopamine signaling may be involved in depression. In addition to DRD2 signaling pathways, dopamine supersensitivity in response to antidepressant treatment is mediated by the activation of the CREB-BDNF signaling pathways in the nucleus accumbens (Guillin et al., 2001; Gershon et al., 2007).

In this study, we used RNA-seq, behavioral tests, western blot (WB), enzyme-linked immunosorbent assay, and co-immunoprecipitation (Co-IP) to comprehensively investigate the role of transcription factor RASD2 in 9-hour fasting on the improvement of depression-like behavior induced by ovariectomy and whether this effect is regulated by DRD2. Considering the significant effects of fasting and estrogen on the BDNF-CREB signaling pathway in the HP and PFC of mice and the fact that RASD2 is enriched in the striatum while interacting with DRD2, in this study, we aimed to investigate the molecular mechanisms involved in the HP, PFC, and striatum.

## MATERIALS AND METHODS

### Animals

Female ICR mice (6–10 weeks, 25 ± 2 g) were purchased from Jilin University (Changchun, China). The mice were kept in plastic cages (25.5 × 15 × 14 cm) under standard laboratory conditions: room temperature 23°C ± 1°C, a 12-hour-light/-dark cycle (7:00 am-7:00 pm light period). Food and water were available ad libitum. Before experiments, mice were randomly assigned to each group. Five mice were housed in 1 cage before surgery and were housed in a single cage after surgery to prevent the mice from biting each other. Transparent cages were used to allow the mice to see each other, and toys were placed in the cages throughout the single-cage rearing period. All experiments were conducted according to the standards set forth in the Laboratory Animal-Guideline for ethical review of animal welfare (GB/T 35892-2018) and under protocols approved by the Institutional Animal Care and Use Committee of Jilin University.

### Experimental Design

**Figure 1.**
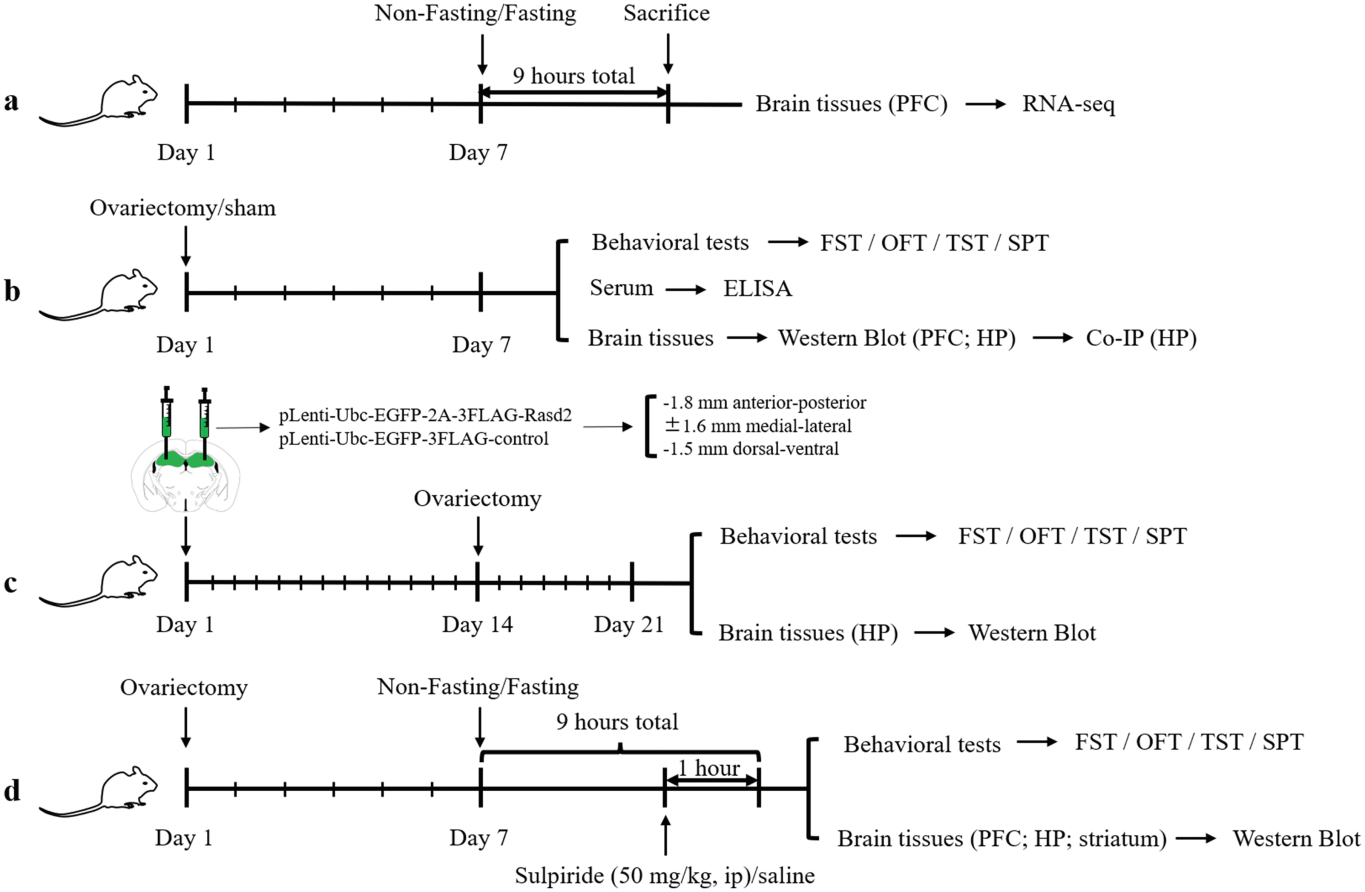
Schematic of experimental design and timeline. Co-IP, co-immunoprecipitation; ELISA, enzyme-linked immunosorbent assay; FST, forced swimming test; HP, hippocampus; OFT, open field test; PFC, prefrontal cortex; SPT, sucrose preference test; TST, tail suspension test.

The experimental design and timeline are shown in Figure 1. To investigate the effect of acute fasting on gene expression in mouse brain, mice were killed after 9-hour fasting or normal diet, and brain tissues (PFC) were dissected and processed for RNA-seq (Figure 1A). To investigate the effect of ovarian removal on depression-like behavior and related changes in protein expression, behavioral tests (FST, n = 13 each group; open field test [OFT; n = 13 each group], tail suspension test [TST; n = 6–7 each group], and sucrose preference test [SPT; n = 8 each group]) and serum (n = 8 each group) and brain tissue (PFC and HP, n = 3–6 each group) extraction were performed 7 days after ovariectomy (Figure 1B).

To explore the effect of *Rasd2* overexpression in the HP on ovariectomy-induced depression, ovariectomy was performed 14 days after injection of the virus (control or *Rasd2*-overexpression), behavioral tests [FST (n = 11–15 each group), OFT (n = 11–15 each group), TST (n = 7–9 each group) and SPT (n = 7–9 each group)] and brain tissue (HP, n = 4–6 each group) dissection was performed 7 days after ovariectomy (Figure 1C).

To investigate the effect of sulpiride (a DRD2 antagonist) on the antidepressant-like effect of 9-hour fasting, sulpiride (50 mg/kg, i.p.; Sigma Aldrich, S8010; dissolved initially in 0.1 M HCl) (Cunha et al., 2012; Donato et al., 2013) was administered after 8-hour fasting. Behavioral tests (FST [n = 11–15 each group], OFT [n = 12–15 each group], TST [n = 7–8 each group], and SPT [n = 8–9 each group]) and brain tissue (PFC, HP, and striatum, n = 3–5 each group) dissection were performed 1 hour after administration (Figure 1D). In all experiments, fasting started at 12:00 am and ended at 9:00 am.

### Surgery

All animals were adapted to the laboratory environment for 3 days before undergoing ovariectomy. The surgical procedure for ovariectomy followed the same procedure described in our previous report (Liu et al., 2012). Briefly, mice were anesthetized with pentobarbital sodium (65 mg/kg, i.p., Dingguo Changsheng Biotechnology, Beijing, China), and the mice were kept in a lateral position. Hair was removed 1 cm horizontally from both sides of the spine, and the skin was disinfected with betadine. A small incision was made parallel to the spine at the intersection of the upper thigh and the lateral spine of the mice, and then the ovaries were removed bilaterally. A week was allowed for recovery before further testing. Sham-operated animals only had incisions without removing the ovaries.

To overexpress *Rasd2* in the HP, a lentiviral expression vector was synthesized by Obio Technology (Shanghai, P.R. China). Viral titers were 4.77*108 particles/mL for pLenti-Ubc-EGFP-2A-3FLAG-Rasd2 and 1.55*109 particles/mL for pLenti-Ubc-EGFP-3FLAG-control. After anesthesia with pentobarbital sodium, mice were placed on the stereotactic frame and the scalp and connective tissue were cut to fully expose the skull. After holes were drilled at the appropriate locations, the virus was microinjected bilaterally into the HP (–1.8 mm anterior-posterior, ±1.6 mm medial-lateral, and –1.5 mm dorsal-ventral from bregma; Figure 1C) at a speed of 0.2 μL/min.

### RNA Isolation, Sequencing, and Bioinformatic Analysis

The animals were decapitated, and the PFC was quickly removed, placed on ice, labeled, and stored in a refrigerator at –80°C for later processing and analysis. Tissue was processed following the instructions of the Trizol kit to extract total RNA and then using RNase-free DNase I to remove genomic DNA. RNA purity and concentration were determined using a Nano Photometer spectrophotometer (IMPLEN, Westlake Village, CA, USA) and a Qubit 2.0 kit. High-quality RNA samples were transported on dry ice to Sangon Biotech (Shanghai, China) for sequencing and testing. The sequencing of the established library was performed with the Illumina HiSeq XTen platform (Illumina, San Diego, CA, USA), and paired-end reads at 150 bp were obtained. The Bioconductor software package was used to correct for multiple testing (false discovery rate cutoff <0.1) and to identify differentially expressed transcripts based on counts per million values. *P <* .05 was considered statistically significant.

### Open Field Test

Mice were placed in the center of an acrylic apparatus (48.8-cm diameter, 16 cm high) (Liu et al., 2012). The floor of the apparatus was divided into 16 equal squares. The test lasted 6 minutes and was recorded with a video camera (DCR-SX83E, Sony, Shanghai, China). Horizontal locomotor activity (the number of grid lines crossing traversed by all 4 paws of the mouse) and vertical locomotor activity (number of times the mouse stood with both forepaws off the ground) were counted by an observer blind to the treatment conditions.

### Forced Swimming Test

Each mouse was individually placed in a cylindrical container (11 cm diameter × 25 cm high), filled with water (12 cm depth), with the water temperature maintained at 25°C ± 1°C (Liu et al., 2012). The test lasted 6 minutes and was recorded with a video camera (DCR-SX83E, Sony). The first 2 minutes of the test were considered adaptation time, and behavior was recorded for the only final 4 minutes of the test. Duration of immobility, swimming, and climbing as well as defecation (number of fecal boli) were determined by an observer blinded to the experimental conditions. Specific discrimination of behavior in the FST (immobility, swimming, and climbing) was according to the criteria previously reported (Cryan et al., 2002). Immobility was defined as having no additional movement other than that necessary to keep the head above the water. Swimming was defined as swimming with the body parallel to the wall. Climbing was characterized by pawing movements oriented at the side of the chamber with the animal oriented perpendicularly to the wall (Cryan et al., 2002).

### Tail Suspension Test

The TST was referred to in previously published articles (Kim et al., 2021; Zhang et al., 2021a). Tape was attached 2 cm from the mouse tail-tip, and the mouse was held in an inverted state with the head approximately 20 cm above the ground with tape. The behavior of the mice within 5 minutes was recorded by a video camera (DCR-SX83E, Sony). The cumulative immobility time (the body of mice was vertically inverted and immobile) during the last 4 minutes was recorded by an observer blind to the treatment conditions.

### Sucrose Preference Test

Mice were trained to acclimate to 1% sucrose solution (two 1% sucrose water bottles per cage) 2 days before the formal test. Mice were water deprived for 12 hours, then 2 weighed water bottles (one 1% sucrose solution and one pure water) were placed in each cage. After 1 hour, all bottles were weighed to calculate sucrose solution and water consumption (Zhang et al., 2021a). Sucrose preference = sucrose solution consumption / (sucrose solution consumption + pure water consumption) * 100%.

### Enzyme-Linked Immunosorbent Assay

Mice were anesthetized and their whiskers were clipped. Blood was collected by retro-orbital bleeding and placed at room temperature for 1 hour, followed by centrifugation at 2000 rpm for 10 minutes. The plasma supernatant was collected and stored at –80°C until use. Assay was performed by recommended protocol of kit (Feiya Biotechnology Co., Ltd, Jiangsu, China). To the wells were added standard or samples and added sample diluent. We then added horseradish peroxidase (HRP)-conjugate reagent to each well and incubated for 60 minutes at 37°C. After washing, chromogen A and B were added to each well and incubated for 15 minutes at 37°C. Finally, stop solution was added to each well. We read optical density at 450 nm by using a microtiter plate reader (Variosbon Flsh, Thermo Scientific, Waltham, MA, USA) within 15 minutes.

### Western Blot

The mice were decapitated, the brain removed, and the PFC, HP, and striatum dissected. Samples from the PFC, HP, and striatum tissue were homogenized in radio immunoprecipitation assay (RIPA) buffer (R0020, Solarbio, Beijing, China) with 1% phenylmethylsulfonyl fluoride (PMSF) solution. The homogenate was centrifuged at 12 000 rpm at 4°C for 20 minutes, and the precipitate was discarded for the removal of insoluble proteins. After mixing with the loading buffer, the samples were placed in boiling water for 5 minutes. Proteins were separated by constant pressure electrophoresis (Bio-Rad, CA, USA) at 110 constant voltages on 10% sodium dodecyl sulphate-polyacrylamide gel electrophoresis (SDS-PAGE) gels. Then the target protein was transferred to polyvinylidene difluoride membranes (100 constant voltages, 1 hour) prior to blocking with 5% skim-milk (dissolved in Tris buffered saline [TBS]) for 2 hours. The membrane was incubated with the primary antibody overnight at 4°C: RASD2 (1:800, rabbit polyclonal; Abcam, Cambridge, UK, #ab67277); BDNF (1:1000, rabbit polyclonal; ABclonal, Wuhan, China, #A16229); CREB (1:1000, rabbit polyclonal; Abcam, #ab32515); p-CREB (1:1000, rabbit polyclonal; CST, Danvers, MA, USA, #9198); Akt (1:1000, rabbit monoclonal; CST, #9272S); DRD2 (1:2000, rabbit polyclonal; Abcam, #ab99446); estrogen receptor alpha (ERα) (1:1000, rabbit polyclonal; Affinity, Danvers, MA, USA, #AF6058); estrogen receptor beta (ERβ) (1:1000, rabbit polyclonal; Affinity, #AF6469); and β-actin (1:2000, mouse monoclonal; Transgen Biotech, Beijing, China, #HC201). After TBST (TBS containing 0.1% Tween-20) washing, the membranes were incubated with secondary antibody (anti-rabbit: 1:1500; ZSBG-Bio, Beijing, China, #ZB2301; anti-mouse: 1:6000; ZSBG-Bio #ZB2305). Then, after incubation for 1 hour, the membranes were washed 3 times with TBST. The target protein signal was detected using enhanced chemiluminescence (ECL) reagent and analyzed with Image J software, version 1.52.

### Co-immunoprecipitation

Co-IP was performed by the recommended protocol of kit manufacturer (Abs955, Absin, Shanghai, China). Firstly, RIPA buffer (R0020, Solarbio) with 1% PMSF solution was added to the collected tissue, and the tissue was homogenized by homogenizer. Then, the samples were centrifuged at 12 000 rpm for 20 minutes at 4°C, and the supernatant was removed for use. Primary antibody (RASD2, RHES-101AP, Fabgennix, Frisco, TX, USA) was added to the samples, while homologous antibodies (Rabbit IgG, abs20035, Absin) from nonspecific immunization were used as controls and the samples were incubated overnight at 4°C. Protein A and G were added to the samples and gently mixed overnight at 4°C then centrifuged at 12 000 rpm for 1 minute to retain the precipitate. Precipitate was washed by wash buffer 3 times. 1*SDS sample buffer was added to resuspend the precipitate, and the sample was held at 95°C–100°C for 5 minutes. All samples were subsequently analyzed by WB.

### Statistical Analysis

All data values are expressed as mean ± SEM and were analyzed by GraphPad Prism Software (version 8.0.1). Student’s *t* test was used to compare means between 2 groups (sham vs ovariectomy; control vs *Rasd2* overexpression). Two-way ANOVA was used to compare the effects of factorial designs (factor 1: fasting; factor 2: sulpiride). When a significant difference was obtained in an ANOVA, post hoc comparisons were performed between means using Tukey’s honestly significant difference test (Tukey’s HSD). *P <* .05 was considered statistically significant. The Shapiro-Wilk test was used to evaluate the normality of the data by SPSS (version 23). Effect size was assessed calculating *η*^*2*^ or Cohen’s *d* as needed by SPSS. Following Cohen (1988), we interpreted estimated *η*^*2*^ and *d* values as follows: *η*^*2*^ = 0.01 small, 0.06 medium, 0.14 large; *d* = 0.2 small, 0.5 medium, 0.8 large.

## RESULTS

### Effect of Acute Fasting on Brain Gene Expression Changes

**Figure 2.**
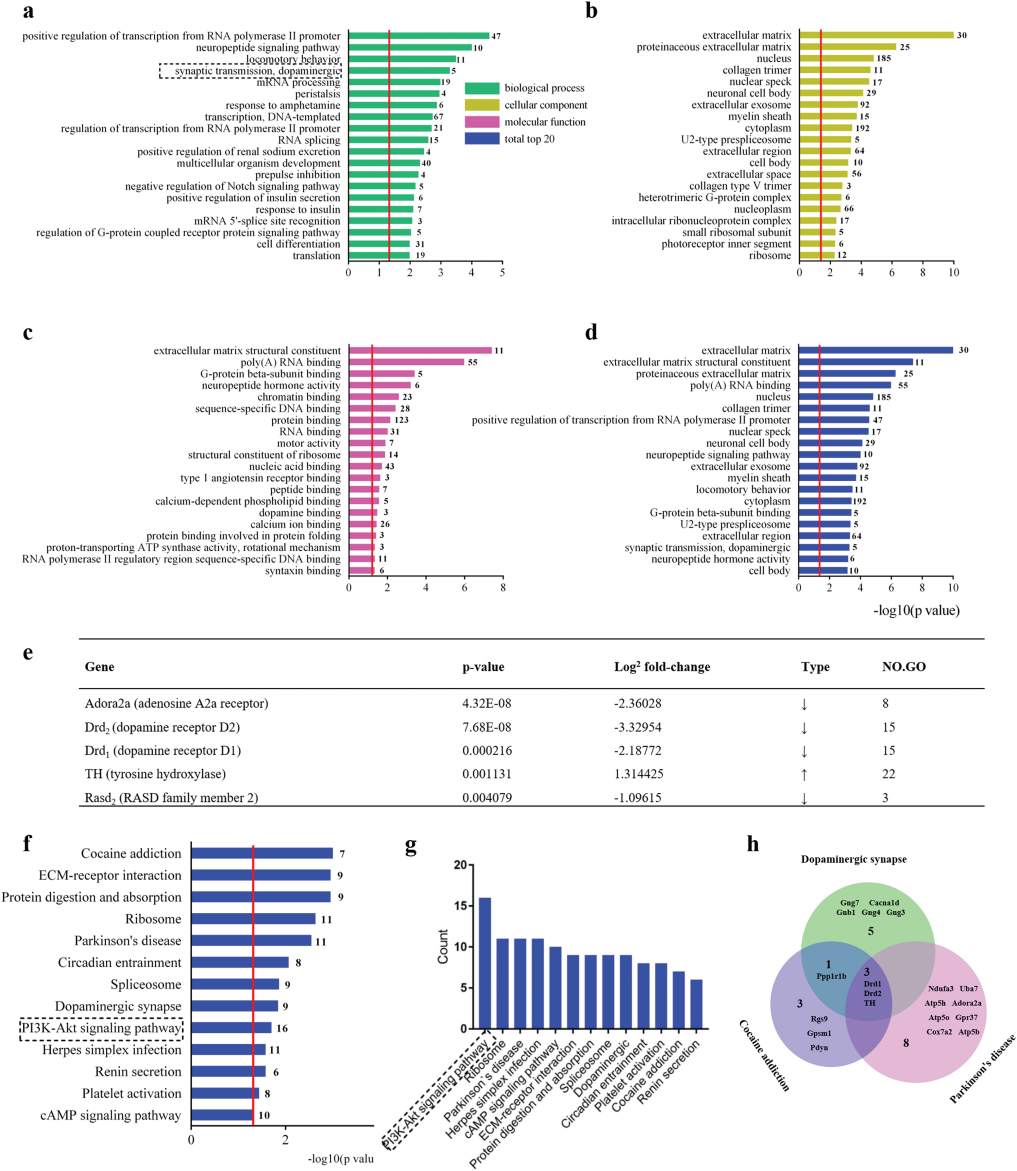
Effects of acute fasting on gene expression in the prefrontal cortex (PFC). (A-D) Differential gene expression in the PFC of mice after 9 hours of acute fasting. Red bars indicate upregulated genes, and blue bars indicate downregulated genes. *P < *.05 was used as the standard to judge whether there is a significant difference in genes. Fold change ≥1.7 is considered to be an upregulated gene, and fold change ≤0.6 is considered to be a downregulated gene. (A) Analysis of the biological process of GO enrichment in the PFC after fasting. (B) Analysis of the composition of GO-enriched cells in the PFC after fasting. (C) Molecular function analysis of GO enrichment in the PFC after fasting. (D) Total analysis of GO enrichment in the PFC after fasting. (E) Differentially expressed genes involved in dopaminergic synaptic transmission. (F–G) KEGG pathway enrichment analysis of genes differentially expressed in the PFC after fasting. (H) Venn diagram of differential gene expression of nervous system-related KEGG pathways in the PFC. The red line represents *P* = .05 and the number at the top of each column represents the number of differentially expressed genes.

Gene expression significantly changed in the PFC as a result of 9-hour fasting. Bioinformatic analysis of the pattern of significantly altered genes is shown in Figure 2 for biological processes (A), cellular components (B), molecular functions (C), and overall functions (D). Figure 2A identified the first 20 biological processes related to differential expressed genes. Among them, dopaminergic synaptic transmission, as well as several biological functions involving dopaminergic neurotransmission, were altered. Figure 2B shows the top 20 cellular components showing altered gene expression. Figure 2C shows the top 20 molecular functions related to differentially expressed genes. Among them, neuropeptide hormone activity, dopamine binding, and syntaxin binding are obviously related to central nervous system functions. Figure 2D shows the top 20 overall results from the analysis of GO enrichment in which the neuronal cell body, neuropeptide signaling pathway, myelin sheath, synaptic transmission (dopaminergic), and neuropeptide hormone activity are related to central nervous system function. The above data suggest that the changes in differentially expressed genes induced by 9-hour fasting may be involved in nerve cell growth and development, hormone regulation, and signal transmission as well as other processes. Further gene function analysis was carried out from the biological process of synaptic transmission (dopaminergic), and genes with significant differences were screened out as shown in Figure 2E: Adenosine A2a receptor (*Adora2a*), *Drd2*, *Drd1*, Tyrosine hydroxylase (*TH*), and *Rasd2*. In the enrichment analysis of the KEGG pathway in the PFC after fasting (Figure 2F–H), the PI3K-Akt pathway has the largest number of differentially expressed genes (16 differentially expressed genes), although the analysis again identified several gene sets related to dopaminergic function (e.g., cocaine addiction, Parkinson’s disease, and dopaminergic synapse), shown in Figure 2H. Ribosomes, Parkinson’s disease, and herpes simplex infection ranked next in this analysis with 11 differentially expressed genes, followed by the cAMP signaling pathway with 10 differentially expressed genes.

### Ovariectomy Induces Depression-Like Behavior and Decreases the Expression of RASD2 and DRD2 in the HP

**Figure 3.**
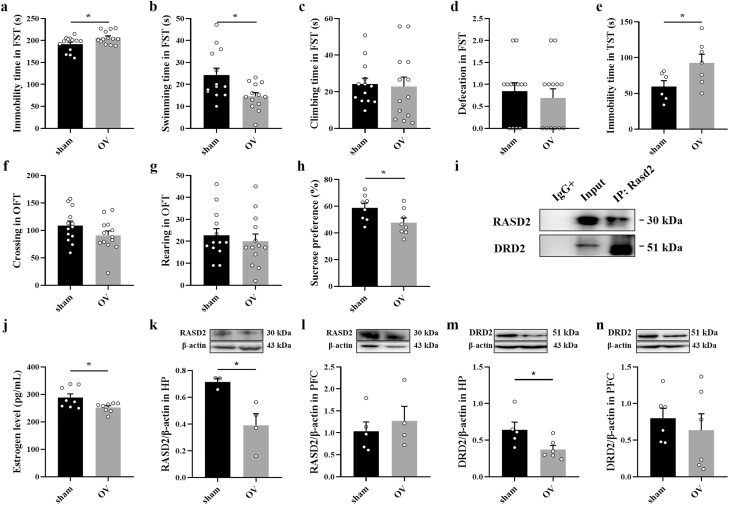
Effects of ovariectomy on depression-like behavior and the expression of RASD Family Member 2 (RASD2) and dopamine D2 receptor (DRD2). Immobility time (A), swimming time (B), climbing time (C), and defecation (D) in the forced swimming test (FST). (E) Immobility time in the tail suspension test (TST). Locomotor behavior (F) and rearing (G) in the open field test (OFT). (H) Sucrose consumption in the sucrose preference test (SPT). (I) A representative image of co-immunoprecipitation in the hippocampus (HP) of ovariectomized mice. (J) Estrogen level in serum. (K–L) The effect of ovarian removal on the expression of RASD2 in the prefrontal cortex (PFC) and HP. (M–N) The effect of ovariectomy on the expression of DRD2 in the PFC and HP. The data are expressed as mean ± SEM. Student’s *t* test, *P < .05 vs sham. OV, ovariectomy.

In the OFT, ovarian removal had no effect on either locomotor activity or rearing (Figure 3F and G). Compared with the sham group, immobility time in the FST and TST significantly increased in ovariectomized mice (FST: t_(24)_ = 2.378, *P* = .0257, *d* = 0.191; TST: t_(11)_ = 2.217, *P* = .0486, *d* = 0.309), and swimming time (t_(24)_ = 2.727, *P* = .0118, *d* = 0.236) and sucrose consumption (t_(14)_ = 2.270, *P* = .0396, *d* = 0.269) significantly decreased (Figure 3A–E and H). In addition, serum estrogen levels were decreased in the ovariectomized mice (t_(14)_ = 2.472, *P* = .0269; Figure 3J). The results of WB showed that ovarian removal reduced RASD2 (t_(5)_ = 3.090, *P* = .0271; Figure 3K) and DRD2 (t_(9)_ = 2.390, *P* = .0406; Figure 3M) expression in the HP but not the PFC (Figure 3L and N). Co-IP showed that RASD2 interacted with DRD2 in the HP of the ovariectomized mice (Figure 3I).

### Overexpression of Rasd2 in the HP Produced Antidepressant-Like Effects and Increased the Expression of DRD2 in Ovariectomized Mice

**Figure 4.**
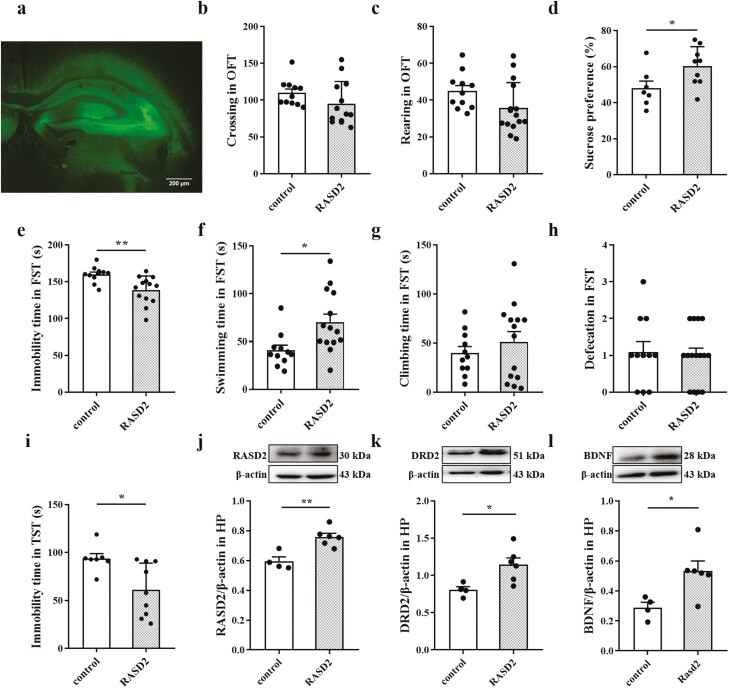
Effects of Rasd2 overexpression on depression-like behavior and the expression of RASD Family Member 2 (RASD2) and dopamine D2 receptor (DRD2). (A) A representative fluorescent image showing green fluorescent protein (GFP) expression in the hippocampus (HP) of virus-injected mouse at 3 weeks after viral delivery. Locomotor behavior (B) and rearing (C) in the open field test (OFT). (D) Sucrose consumption in the sucrose preference test (SPT). Immobility time (E), swimming time (F), climbing time (G), and defecation (H) in the forced swimming test (FST). (I) Immobility time in the tail suspension test (TST). Figures represent changes in the protein expression of RASD2 (J), DRD2 (K), and brain-derived neurotrophic factor (BDNF) (L) in the HP of mice. The data are expressed as mean ± SEM. Student’s *t* test, *P < .05, **P < .01.

As shown in Figure 4e and F, *Rasd2* overexpression in the HP decreased immobility time (t_(22)_ = 3.249, *P* = .0037, *d* = 0.324) and increased the swimming time (t_(23)_ = 2.749, *P* = .0114, *d* = 0.247) of ovariectomized mice in the FST while having no effects on other behavioral measures in the FST or OFT (Figure 4B, C, G, and H). In addition, *Rasd2* overexpression increased sucrose consumption (t_(14)_ = 2.252, *P* = .0409, *d* = 0.266; Figure 4D) and reduced the immobility time in the TST (t_(14)_ = 2.832, *P* = .0133, *d* = 0.364; Figure 4I). Figure 4J–L show that overexpression of *Rasd2* increased RASD2 (t_(8)_ = 4.174, *P* = .0031), DRD2 (t_(8)_ = 2.803, *P* = .0231), and BDNF (t_(8)_ = 2.796, *P* = .0234) expression in the HP.

### Sulpiride Reversed the Alleviating Effects of Acute Fasting on Depression-Like Behaviors

**Figure 5.**
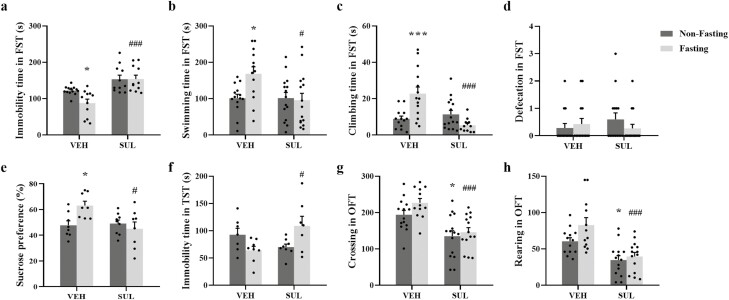
Effects of sulpiride on the antidepression-like effect of fasting. Immobility time (A), swimming time (B), climbing time (C), and defecation (D) in the forced swimming test (FST). (E) Sucrose consumption in the sucrose preference test (SPT). (F) Immobility time in the tail suspension test (TST). Locomotor behavior (G) and rearing (H) in the open field test (OFT). The data are expressed as mean ± SEM. Two-way ANOVA with Tukey’s honestly significant difference (HSD), *P < .05 vs vehicle (VEH):non-fasting; ^#^*P < *.05, ^###^*P < *.001 vs VEH:fasting. SUL, sulpiride.

As shown in Figure 5A, fasting decreased immobility time in the FST in vehicle-treated ovariectomized mice (F_sulpiride(1,42)_ = 27.60, *P*  *<* .0001, *η²* = 0.396; F_fasting(1,42)_ = 3.351, *P* = .0743, *η²* = 0.074; F_sulpiride×fasting(1,42)_ = 3.543, *P* = .0667, *η²* = 0.078). Thus, there was a significant post hoc Tukey’s HSD comparison between the fasting and non-fasting vehicle-treated groups (*P* = .0495). Sulpiride reversed the effect of fasting (*P* *<* .0001). As shown in Figure 5B and C, fasting increased swimming time (F_sulpiride(1,53)_ = 4.748, *P* = .0338, *η²* = 0.082; F_fasting(1,53)_ = 3.419, *P* = .0700, *η²* = 0.060; F_sulpiride×fasting(1,53)_ = 4.856, *P* = .0319, *η²* = 0.084; Tukey’s HSD: *P* = .0355) and climbing time (F_sulpiride(1,50)_ = 9.633, *P* = .0031, *η²* = 0.162; F_fasting(1,50)_ = 2.639, *P* = .1106, *η²* = 0.050; F_sulpiride×fasting(1,50)_ = 16.99 *P* = .0001, *η²* = 0.254; Tukey’s HSD: *P* = .0009) in the vehicle-treated mice. Sulpiride reduced swimming time (*P* = .0173) and climbing time (*P < *.0001) in the fasted mice.

As shown in Figure 5E, fasting increased sucrose consumption in the vehicle-treated mice (F_sulpiride(1,29)_ = 4.837, *P* = .0360, *η²* = 0.143; F_fasting(1,29)_ = 2.214, *P* = .1476, *η²* = 0.071; F_sulpiride×fasting(1,29)_ = 6.540, *P* = .0160, *η²* = 0.184; Tukey’s HSD: *P* = .0403), and sulpiride treatment eliminated the effects of acute fasting on sucrose consumption (*P* = .0124). As shown in Figure 5F, sulpiride reduced immobility time in the TST in the fasted mice (F_sulpiride(1,26)_ = 9.889, *P* = .3292, *η²* = 0.037; F_fasting(1,26)_ = 0.2075, *P* = .6525, *η²* = 0.008; F_sulpiride×fasting(1,26)_ = 9.209, *P* = .0054, *η²* = 0.262). Tukey’s HSD showed that sulpiride treatment eliminated the effects of fasting on immobility time (*P* = .0397).

Figure 5G and H show that sulpiride decreased locomotor activity (F_sulpiride(1,51)_ = 27.64, *P < *.0001, *η²* = 0.066; F_fasting(1,51)_ = 2.690, *P* = .1071, *η²* = 0.363; F_sulpiride×fasting(1,51)_ = 0.6708, *P* = .4166, *η²* = 0.023) or rearing (F_sulpiride(1,49)_ = 25.90, *P*  *<* .0001, *η²* = 0.346; F_fasting(1,49)_ = 3.962 *P* = .0521, *η²* = 0.075; F_sulpiride×fasting(1,49)_ = 1.690, *P* = .1997, *η²* = 0.033) in both fasting- and non-fasting–treated mice. Tukey’s HSD showed that there were significant differences after sulpiride treatment in non-fasting–treated mice (*P* = .0112) and in fasting-treated mice (*P* = .0006) on locomotor activity. And there were significant differences after sulpiride treatment in non-fasting–treated mice (*P* = .0445) and in fasting-treated mice (*P* = .0003) on rearing. It should be noted that sulpiride reduced locomotor activity and rearing in both fasted- and non-fasted mice, which may be an indication of general motor impairing effects.

### **Sulpiride Reversed the Fasting-Induced Increase in RASD2, ER** *β*  **, Activation of DRD2-Linked, and CREB-BDNF Signaling Pathway**

**Figure 6.**
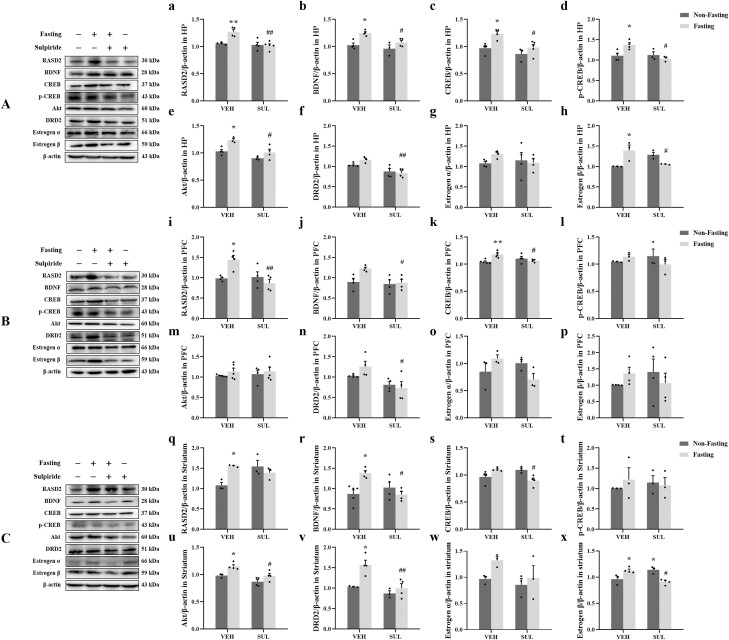
Effect of sulpiride (SUL) on fasting-induced changes in protein expression. Figures represent the changes in the protein expression of RASD Family Member 2 (RASD2), brain-derived neurotrophic factor (BDNF), cAMP-response element binding protein (CREB), phospho-CREB (p-CREB), protein kinase B (Akt), dopamine D2 receptor (DRD2), estrogen receptor α (ERα), and estrogen receptor β (ERβ) in the hippocampus (HP) (A, a–h), prefrontal cortex (PFC) (B, i–p), and striatum (C, q–x) in ovariectomized mice. The data are expressed as mean ± SEM (n = 3–5). Two-way ANOVA with Tukey’s honestly significant difference (HSD), *P  *<* .05 vs ovariectomy (OV), **P  *<* .01 vs vehicle (VEH):non-fasting; ^#^*P*  *<* .05, ^##^*P*  *<* .01 vs VEH:fasting.

The effects of sulpiride on fasting-induced changes in protein expression in the HP are shown in Figure 6A (A–H). ANOVA showed that fasting increased RASD2 in vehicle-treated mice but not sulpiride-treated mice (F_sulpiride(1,13)_ = 14.50, *P* = .0022; F_fasting(1,13)_ = 7.522, *P* = .0168; F_sulpiride×fasting(1,13)_ = 9.363, *P* = .0091). Tukey’s HSD confirmed that there was a significant effect of fasting in vehicle-treated mice (*P* = .0072), and the effect of fasting was reversed by sulpiride (*P* = .0012). A similar pattern was seen for BDNF (F_sulpiride(1,11)_ = 8.140, *P* = .0157; F_fasting(1,11)_ = 15.95, *P* = .0021; F_sulpiride×fasting(1,11)_ = 1.615, *P* = .2301), CREB (F_sulpiride(1,12)_ = 9.909, *P* = .0084; F_fasting(1,12)_ = 11.40, *P* = .0055; F_sulpiride×fasting(1,12)_ = 1.660, *P* = .2219), and p-CREB (F_sulpiride(1,10)_ = 6.707, *P* = .0270; F_fasting(1,10)_ = 2.060, *P* = .1818; F_sulpiride×fasting(1,10)_ = 8.168, *P* = .0170) in the ANOVA. And Tukey’s HSD showed that there was a significant effect of fasting in vehicle-treated mice (BDNF: *P* = .0118; CREB: *P* = .0256; p-CREB: *P* = .0353). Again, the effect of fasting was reversed by sulpiride (BDNF: *P* = .0479; CREB: *P* = .0252; p-CREB: *P* = .0142). A similar pattern was also seen for Akt (F_sulpiride(1,10)_ = 16.12, *P* = .0025; F_fasting(1,10)_ = 12.74, *P* = .0051; F_sulpiride×fasting(1,10)_ = 1.257, *P* = .2884) and DRD2 (F_sulpiride(1,11)_ = 20.74, *P* = .0008; F_fasting(1,11)_ = 0.6827, *P* = .4262; F_sulpiride×fasting(1,11)_ = 2.253, *P* = .1615) as shown in the 2-way ANOVA. Tukey’s HSD showed that there was a significant effect of fasting in vehicle-treated mice (Akt: *P* = .0458), and this effect was normalized by sulpiride (Akt: *P* = .0273; DRD2: *P* = .0078). There were no significant effects of fasting or sulpiride on ERα expression. However, a similar pattern was observed for ERβ as for some of the other measures (F_sulpiride(1,8)_ = 0.1456, *P* = .7127; F_fasting(1,8)_ = 1.178, *P* = .3093; F_sulpiride×fasting(1,8)_ = 17.81, *P* = .0029). Tukey’s HSD showed that there was a significant effect of fasting in vehicle-treated mice (*P* = .0233). Sulpiride reduced ERβ in fasted mice (*P* = .0465) but not in unfasted mice.

WB results for the PFC are shown in Figure 6B (I–P). Acute fasting increased RASD2 expression in the PFC, and this effect was reversed by sulpiride (F_sulpiride(1,12)_ = 7.826, *P* = .0161; F_fasting(1,12)_ = 2.554, *P* = .1360; F_sulpiride×fasting(1,12)_ = 9.802, *P* = .0087). Tukey’s HSD showed that there was a significant effect of fasting in vehicle-treated mice (*P* = .0260). Sulpiride decreased RASD2 expression in fasted mice (*P* = .0059) but not unfasted mice. A similar pattern was seen for BDNF (F_sulpiride(1,12)_ = 5.896, *P* = .0318; F_fasting(1,12)_ = 4.782, *P* = .0493; F_sulpiride×fasting(1,12)_ = 3.273, *P* = .0955) and CREB (F_sulpiride(1,14)_ = 1.278, *P* = .2773; F_fasting(1,14)_ = 3.412, *P* = .0860; F_sulpiride×fasting(1,14)_ = 11.73, *P* = .0041) in the 2-way ANOVA. Tukey’s HSD showed that there was a significant effect of fasting in vehicle-treated mice (CREB: *P* = .0070) and a significant effect of sulpiride treatment in fasted mice (BDNF: *P* = .0477; CREB: *P* = .0279). DRD2 expression in the PFC was also increased by fasting, and this effect was normalized by sulpiride (F_sulpiride(1,12)_ = 11.54, *P* = .0053; F_fasting(1,12)_ = 0.4593, *P* = .5108; F_sulpiride×fasting(1,12)_ = 2.068, *P* = .1759). Tukey’s HSD showed that there was significant effect of sulpiride in fasted mice (*P* = .0228) in the PFC. ANOVA did not find significant effects of sulpiride treatment or fasting on the expression of p-CREB, Akt, ERα, or ERβ.

WB results for the striatum are shown in Figure 6C (Q–X). Fasting increased expression of RASD2 in the striatum, but this effect was not reversed by sulpiride (F_sulpiride(1,8)_ = 2.465, *P* = .1551; F_fasting(1,8)_ = 2.847, *P* = .1300; F_sulpiride×fasting(1,8)_ = 11.22, *P* = .0101). Tukey’s HSD showed that there was a significant effect of fasting in the vehicle-treated groups (*P* = .0303). Expression of BDNF (F_sulpiride(1,13)_ = 3.417, *P* = .0874; F_fasting(1,13)_ = 2.824, *P* = .1167; F_sulpiride×fasting(1,13)_ = 11.46, *P* = .0049) and CREB (F_sulpiride(1,11)_ = 0.7837, *P* = .3949; F_fasting(1,11)_ = 0.5026, *P* = .4931; F_sulpiride×fasting(1,11)_ = 14.80, *P* = .0027) in the striatum were also increased by fasting, and this effect was eliminated by sulpiride. Tukey’s HSD showed that there was a significant effect of fasting in vehicle-treated mice (BDNF: *P* = .0129) but not sulpiride-treated mice. Sulpiride administration decreased the expression of BDNF in fasted mice (BDNF: *P* = .0148; CREB: *P* = .0226). Expression of Akt (F_sulpiride(1,11)_ = 14.41, *P* = .0030; F_fasting(1,11)_ = 13.38, *P* = .0038; F_sulpiride×fasting(1,11)_ = 0.6597, *P* = .4339) and DRD2 (F_sulpiride(1,10)_ = 13.54, *P* = .0042; F_fasting(1,10)_ = 10.81, *P* = .0082; F_sulpiride×fasting(1,10)_ = 4.116, *P* = .0700) in the striatum was also increased by fasting and normalized by sulpiride. Tukey’s HSD found significant effects of fasting in vehicle-treated mice (Akt: *P* = .0470; DRD2: *P* = .0165), and sulpiride decreased Akt (*P* = .0264) and DRD2 (*P* = .0065) expression in fasted mice. Fasting also increased ERβ expression in the striatum, and this effect was reversed by sulpiride treatment (F_sulpiride(1,10)_ = 0.3373, *P* = .5743; F_fasting(1,10)_ = 0.8156, *P* = .3877; F_sulpiride×fasting(1,10)_ = 30.44, *P* = .0003). Tukey’s HSD showed that fasting increased ERβ expression in the striatum in vehicle-treated mice (*P* = .0362), and sulpiride reduced ERβ expression only in fasted mice (*P* = .0042). In non-fasted mice, sulpiride increased the expression of ERβ (*P* = .0360). There were no significant effects of sulpiride treatment or fasting on p-CREB or ERα levels in the striatum.

## DISCUSSION

In the present study, we found that 9-hour fasting altered differential gene expression in the PFC of ovariectomized mice. The results of GO enrichment analysis and KEGG pathway enrichment analysis of differentially expressed genes showed that fasting affected genes related with dopaminergic signaling, including *Drd2*, *Drd1*, *TH*, and *Rasd2*. A study also found that calorie restriction causes dopaminergic dysregulation in female mice (Carlin et al., 2016). In addition, calorie restriction delays the age-related or diabetes-related loss of DRD2 in rat brain (Roth et al., 1984; Thanos et al., 2008; de Leeuw van Weenen et al., 2011). Our results are consistent with the above studies showing that the molecular mechanisms of fasting on depression may be closely linked to dopamine.

To further explore the molecular mechanisms underlying the effects of fasting in ovariectomized mice, *Drd2* and *Rasd2* were selected from the RNA-seq study for further study. Interestingly, in our studies, RASD2 protein was decreased in ovariectomized mice in the HP but not in the PFC. These results indicate that the depression model established by ovariectomy induces the downregulation of RASD2 in the HP (but not in the PFC), and the downregulation of RASD2 expression in the HP is one of the pathogeneses of depression. Although *Rasd2* has been reported to be a common regulator of fasting and estrogen in the PFC (Wang et al., 2019), there may be other regulators involved in ovariectomized mice. It has been reported that short-term fasting increases autophagy in cortical neurons (Alirezaei et al., 2010), and overexpression of *Rasd2* can also activate autophagy (Mealer et al., 2014). However, whether short-term fasting further increases autophagy through *Rasd2* is still unknown, and this should be investigated in further studies. In addition, ovariectomy also reduced DRD2 expression in the HP.

Considering that there is a high density of DRD2 binding site in dorsal HP (Charuchinda et al., 1987; Edelmann and Lessmann, 2018), lentivirus vectors was microinjected into the dorsal HP to achieve *Rasd2* overexpression in the HP of ovariectomized mice. *Rasd2* overexpression in ovariectomized mice significantly decreased immobility in the FST and TST and increased swimming time and sucrose consumption, indicative of antidepressant effects. No effects were observed in the OFT, showing that the effects of *Rasd2* overexpression were behaviorally specific and not just the result of elevated spontaneous activity. These results indicate that *Rasd2* and DRD2 are fundamentally involved in ovariectomy-induced depression. Previous studies have shown that antidepressants work on catecholaminergic systems selectively increase climbing behavior, whereas antidepressants targeting serotonergic systems selectively increase swimming behavior (Cryan et al., 2005; Slattery and Cryan, 2012). In addition, emotional animals defecate more than non-emotional animals (Broadhurst, 1957; Craft et al., 2010). In our study, *Rasd2* overexpression primarily increased swimming, with only modest effects on climbing and defecating behavior. Whether these effects involve dopamine or dopamine interactions with other monoaminergic systems is uncertain. In addition, *Rasd2* overexpression in the HP of ovariectomized mice significantly increased DRD2 expression in the HP. Studies have found that *Rasd2* affects DRD2-dependent activity (Sciamanna et al., 2015) and also regulates striatal-dependent behaviors in a gender-specific manner (Ghiglieri et al., 2015). These results indicate that *Rasd2* and DRD2 are likely to be involved in the molecular mechanisms underlying depressive-like symptoms induced by ovariectomy.

The role of RASD2 in DRD2-mediated antidepressant-like effects of 9-hour fasting based on ovariectomized mice was then examined. Immobility time in the FST was increased and sucrose consumption decreased, indicative of a depressive profile, and fasting significantly reversed the changes of depression-like behaviors. Moreover, DRD2 antagonists blocked the antidepressant-like effects of fasting. Antidepressant-like effects of fasting on climbing and swimming in the FST may involve dopamine or its interaction with other monoaminergic systems. This finding is consistent with previous studies showing that sulpiride antagonizes antidepressant effects on immobility (Borsini et al., 1988; Donato et al., 2013). Importantly, the present study suggests that DRD2 mediates the reduction in immobility time induced by fasting as well. Nonetheless, RASD2 and DRD2 appear to be intimately related in the effects of ovariectomy and fasting. Firstly, the antidepressant-like effect of 9-hour fasting involving DRD2 was shown to be closely related to RASD2 expression. Fasting increased the expression of RASD2 in the PFC, HP, and striatum, and DRD2 antagonists reversed this increase in RASD2 levels induced by fasting. Similarly, fasting increased DRD2 expression in the PFC and HP. These results suggest that the reduction in immobility caused by fasting is likely to be caused by regulating the expression of DRD2 and RASD2.

Studies have found that *Rasd2* deficiency leads to abnormal excitatory responses of cholinergic interneurons to activation of DRD2 receptors. Furthermore, PI3K inhibitors rescue the abnormal DRD2 response in *Rasd2* knockout mice, and it has been found RASD2 acts as a bridge between PI3K and Akt signaling pathways (Subramaniam et al., 2011; Bang et al., 2012; Harrison et al., 2013; Lee et al., 2015). Fasting was shown to activate the PI3K-Akt pathway in the KEGG pathway analysis and lead to a decrease in Akt expression, and sulpiride treatment reverses the fasting-induced increases in Akt expression. These results indicate that changes in Akt expression may also participate in the reduction of immobility time mediated by DRD2.

The PI3K-Akt pathway is a DRD2-linked signaling pathways that has been linked to the pathogenesis of mood disorders and BDNF-mediated neuroprotection (Cao et al., 2019; Huang et al., 2021). Consistent with previous studies (Cui et al., 2018; Wang et al., 2019), in our studies, fasting increased CREB and BDNF expression in the PFC and HP of ovariectomized mice. Moreover, DRD2 antagonist antagonized fasting-induced increases in CREB and BDNF expression, indicating that the CREB-BDNF signaling pathway is likely to play a role in the antidepressant-like effects of fasting mediated by DRD2. Therefore, RASD2 may produce antidepressant-like effects by regulating the expression of Akt and further affecting the CREB-BDNF signaling pathway.

It has been reported that the mouse plasma levels of estrone, estradiol, and estriol were reduced 1 week after ovariectomy, and estradiol and estriol levels in plasma were similar between 1 week and 3 months post-ovariectomy and 17β-estradiol treatment (Zhang et al., 2019). In the present study, ovariectomy induced an increase in immobility in the FST at 1 week after ovariectomy, similar to previous findings (Estrada-Camarena et al., 2011). In previous studies, caloric restriction produced estrogen-like effects (Bigsby et al., 1997) and increased estrogen levels; moreover, there was an additive antidepressant-like effect of fasting and estrogen in ovariectomized mice (Wang et al., 2019). Fasting increased ERβ expression in the HP and striatum, an effect antagonized by sulpiride. 17 β-Estradiol has no effect on immobility in ERβ knockout mice, but not ERα knockout mice in the FST (Rocha et al., 2005). These studies indicate that the increase in immobility time of ovariectomized mice is mainly related to ERβ. Moreover, the present study suggests that ERβ is involved in the effects mediated by DRD2 on the antidepressant-like effects of fasting, although the connection between RASD2, DRD2, and ERβ remains to be fully elucidated.

## CONCLUSION

**Figure 7.**
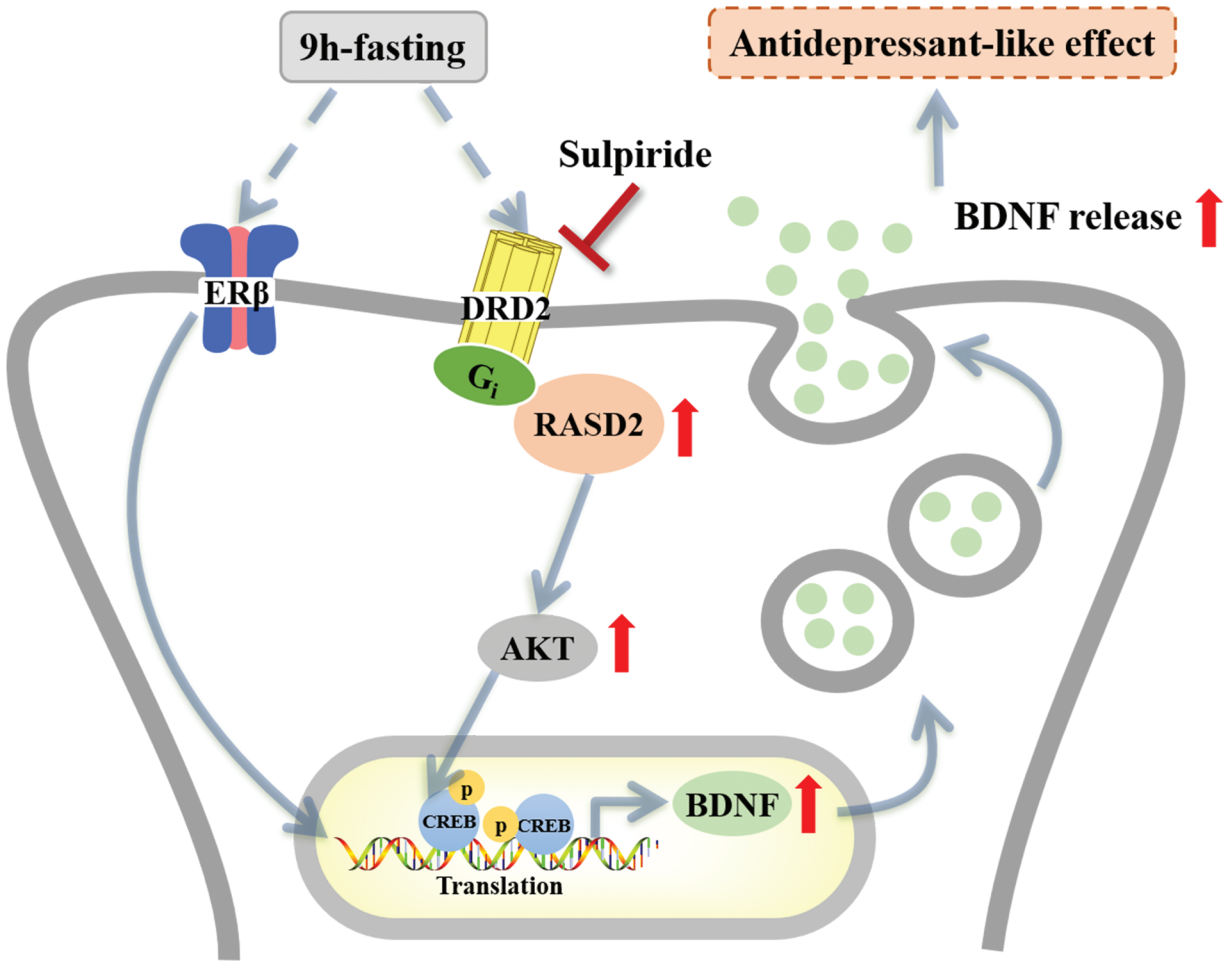
A schematic diagram of the effect of RASD Family Member 2 (RASD2) in dopamine D2 receptor (DRD2)-mediated antidepressant-like effects produced by acute fasting in the hippocampus (HP). Akt, protein kinase B; BDNF, brain-derived neurotrophic factor; CREB, cAMP-response element binding protein; ERβ, estrogen receptor β; Gi, inhibitory adenylate cyclase g protein.

In summary, *Rasd2* plays a role in depression-like behavior induced by ovariectomy, and this role is related to the regulation of DRD2. Nine-hour fasting has antidepressant-like effects in ovariectomized mice and upregulates the expression of RASD2, DRD2, CREB-BDNF, Akt, and ERβ (Figure 7). Moreover, these effects are blocked by DRD2 antagonists. *Rasd2* can therefore be postulated to be a potential therapeutic target for depression and perhaps also a potential predictive marker for depression. Finally, dopamine receptor-mediated gene regulation in antidepressant-like effects of acute fasting also provides new ideas for the treatment of depression. However, whether fasting has similar therapeutic effects on patients with depression and the conditions for implementing fasting (such as the specific time point and duration of fasting) need to be further explored in clinical studies.

## Data Availability

The data underlying this article will be shared on reasonable request to the corresponding author.
