# Corrigendum to: Rasd2 Mediates Acute Fasting-Induced Antidepressant-Like Effects via Dopamine D2 Receptor Activation in Ovariectomized Mice

**DOI:** 10.1093/ijnp/pyad015

**Published:** 2023-04-01

This is a corrigendum to: Ziqian Cheng, Fangyi Zhao, Jingjing Piao, Ranji Cui, Bingjin Li, Rasd2 Mediates Acute Fasting-Induced Antidepressant-Like Effects via Dopamine D2 Receptor Activation in Ovariectomized Mice, *International Journal of Neuropsychopharmacology*, Volume 26, Issue 3, March 2023, Pages 217–229, https://doi.org/10.1093/ijnp/pyac082

In the originally published version of this manuscript, Chaohe Zhang was erroneously included in the list of authors.

This error has now been corrected.

